# MORE ADULT AMERICANS ARE AT NUTRITIONAL RISK SINCE THE COVID-19 PANDEMIC

**DOI:** 10.1093/geroni/igad104.2265

**Published:** 2023-12-21

**Authors:** Lillie Monroe-Lord, Azam Ardekani, Cassidy Whitman

**Affiliations:** University of the Dustrict of Columbia, Washington, District of Columbia, United States; University of the District of Columbia, Washington, District of Columbia, United States; University of the District of Columbia, Washington, District of Columbia, United States

## Abstract

The nutritional status of individuals has changed since COVID-19 pandemic due to disruptions in social, economic, and psychological factors. This study determines how nutritional status has changed since COVID-19 among American adults. The second aim is to examine which age range group was at a higher risk of nutritional status due to the pandemic. The study design was cross-sectional, with 10,035 participants aged 40-100 who answered a survey distributed by Qualtrics. Nutritional status was measured twice before and since the COVID-19 pandemic by the Dietary Screening Tool (DST). The total DST score was calculated for each participant, which categorized them into three different groups, including “at risk,” “possible risk,” and “not at risk.” The results revealed that the mean DST score before the pandemic was 56.04 (IQR: 48.00–65.00); this score significantly decreased since COVID-19 to 55.54 (IQR: 47.00–64.00) (p <.001). Of those participants who were not at risk before COVID-19, 28.5% became either at risk or at possible risk since COVID-19. Of those participants who were at possible risk before COVID-19, 21% are now at risk since COVID-19. Almost 60% of the younger group (40-60) were at risk before COVID-19 compared to 54.8% of participants aged 61-80 and 45.8% of older participants (81-100). These percentages increased by about 2% since COVID-19 among all three age groups. As a good nutritional status reduce the risk of severe illness, these findings can help policymakers to develop heath-protecting behavior sessions against future pandemics to manage crises.

